# Aspirin Inhibits the In Vitro Adipogenic Differentiation of Human Adipose Tissue-Derived Stem Cells in a Dose-Dependent Manner

**DOI:** 10.3390/ijms26020853

**Published:** 2025-01-20

**Authors:** Sarah Funke, Paul Severin Wiggenhauser, Anna Grundmeier, Benedikt Fuchs, Konstantin Koban, Wolfram Demmer, Riccardo E. Giunta, Constanze Kuhlmann

**Affiliations:** Division of Hand Surgery, Plastic Surgery and Aesthetic Surgery, University Hospital, LMU Munich, Ziemssenstraße 5, 80336 Munich, Germany; sarah.funke@campus.lmu.de (S.F.); severin.wiggenhauser@med.uni-muenchen.de (P.S.W.); anna.grundmeier@campus.lmu.de (A.G.); benedikt.fuchs@med.uni-muenchen.de (B.F.); konstantin.koban@med.uni-muenchen.de (K.K.); wolfram.demmer@med.uni-muenchen.de (W.D.); riccardo.giunta@med.uni-muenchen.de (R.E.G.)

**Keywords:** acetylsalicylic acid, ASA, adipogenic differentiation, fat tissue engineering, regenerative medicine

## Abstract

Aspirin (ASA) is one of the most used medications worldwide and has shown various effects on cellular processes, including stem cell differentiation. However, the effect of ASA on adipogenesis of adipose tissue-derived stem cells (ASCs) remains largely unknown. Considering the potential application of ASCs in regenerative medicine and cell-based therapies, this study investigates the effects of ASA on adipogenic differentiation in human ASCs. ASCs were exposed to varying concentrations of ASA (0 µM, 400 µM, and 1000 µM) and evaluated for changes in morphology, migration, and adipogenic differentiation. While ASA exposure did not affect self-renewal potential, migration ability, or cell morphology, it significantly reduced lipid vacuole formation at 1000 µM after 21 days of adipogenic differentiation (*p* = 0.0025). This visible inhibition correlated with decreased expression of adipogenic markers (PPARG, ADIPOQ, and FABP4) and the proliferation marker MKi67 under ASA exposure in comparison to the control (ns). Overall, the findings demonstrate that ASA inhibits adipogenic differentiation of human ASCs in a dose-dependent manner in vitro, contrasting its known role in promoting osteogenic differentiation. This research highlights ASA’s complex effects on ASCs and emphasizes the need for further investigation into its mechanisms and potential therapeutic applications in obesity and metabolic diseases. The inhibitory effects of ASA on adipogenesis should be considered in cell-based therapies using ASCs.

## 1. Introduction

White adipose tissue, a dynamic and metabolically active organ, not only serves as a subcutaneous energy storage depot but also exerts endocrine function through the secretion of cytokines [1]. It contains a diverse population of cells, with mature adipocytes being the primary cellular component. Other cellular components comprise preadipocytes, pericytes, fibroblasts, smooth muscle cells, endothelial cells, hematopoietic cells, macrophages, mature immune cells such as B and T cells, and myeloid cells [2]. Additionally, adipose tissue-derived stem cells (ASCs) are found within white adipose tissue and can be isolated from the stromal vascular fraction (SVF), which is obtained by processing the adipose tissue through either enzymatic or mechanical methods [3]. First described in 2002, ASCs exhibit remarkable plasticity and can differentiate into multiple cell lineages, including the adipogenic pathway, as well as chondrogenic, osteogenic, endothelial, myogenic, cardiomyogenic, and neuronal lineages [1,4,5,6]. Owning to their stem cell properties and ease of isolation through minimally invasive liposuction, ASCs have emerged as a promising cell source for innovative therapeutic approaches to tissue repair and regeneration [7,8].

Within adipose tissue, ASCs are situated in the “adiponiche”, a specialized and finely tuned microenvironment. This niche contributes to the quiescence of ASCs, preserves their stemness, and regulates their proliferation and differentiation. Furthermore, it supports physiological adipocyte turnover and maintains energy balance [9]. Disbalance or damage of the adiponiche creates a dysfunctional microenvironment, contributing to the pathological changes (adiposopathy) seen in metabolic disease and obesity [9,10]. During adipogenesis, stem cells go through several phases (commitment and terminal differentiation) and stages, starting from the preadipocyte stage, through early adipocytes to mature adipocytes, with multiple signaling pathways regulating this process [11,12,13]. As Chen et al. summarized in 2016, these regulatory pathways include TGFβ/BNP signaling, the WNT, the Notch pathway, and Hedgehog signaling, which control the differentiation lineage of MSCs [12,14,15,16]. Particularly noteworthy are the C/EBPα and the WNT, which are thought to play a key role in the differentiation process [13,17,18,19]. When undergoing adipogenesis, ASCs increasingly express adipogenic markers, including peroxisome proliferator-activated receptor gamma (*PPARγ*), adiponectin (*ADIPOQ*), and fatty acid binding protein 4 (*FABP4*) [11]. Understanding the regulatory mechanisms and external influences that govern adipogenic differentiation of ASCs is vital, not only for the development and engineering of connective tissues but also for unraveling the pathophysiology of obesity, metabolic disorders, and related diseases. Furthermore, investigating the impact of pharmacological agents on adipogenesis and stemness maintenance offers a promising avenue for therapeutic intervention.

In this regard, acetylsalicylic acid (ASA), commonly known as “aspirin”, a name given by the company Bayer in 1899, has previously demonstrated the potential to impact some characteristics of mesenchymal stem cell (MSC) sources including ASCs in vitro and in vivo [20,21,22,23,24]. ASA, one of the oldest chemically defined substances, originated from willow bark and was utilized for medicinal purposes since Mesopotamian times, around 6000 years ago [25]. In 1897, Felix Hoffmann, a young pharmaceutical chemist at Bayer, successfully acetylated the phenol group of salicylic acid by refluxing it with acetic anhydride, thus first obtaining ASA, the form of the drug we use today. Like many drugs, the therapeutic effects of ASA—such as its anti-inflammatory, analgesic, and thrombosis-preventive properties—were observed empirically long before its molecular mechanism was understood [26]. In 1971, John R. Vane proposed that the mechanism of action for ASA and other NSAIDs involves the dose-dependent inhibition of prostaglandin biosynthesis. This discovery, along with his subsequent identification of prostacyclin, earned him the Nobel Prize for Medicine in 1982 [27,28]. The primary target of ASA is an irreversible inhibition of COX-1 and modulation of COX-2 by acetylation of a serine residue in the active center of the AA binding channels [29]. This reduces prostaglandin synthesis, leading to analgesic and antipyretic effects (COX-2) and inhibits platelet aggregation by reducing thromboxane A2 synthesis (COX-1) [28,30,31]. More than 125 years after its industrial synthesis, ASA remains a focal point in scientific research, potentially extending its clinical applications beyond cardio- and cerebrovascular risk prevention and its use as an NSAID [32]. Ongoing trials explore its potential in cancer chemoprevention, including its ability to reduce the incidence of several solid cancers, particularly gastrointestinal carcinomas, and its significant anti-arteriosclerotic effects in preventing myocardial infarction [33,34,35,36]. Additionally, ASA also alleviates the symptoms of metabolic syndrome related to obesity, suggesting it may influence adipose tissue metabolism [37,38,39]. Despite these numerous beneficial properties, the widespread and unrestricted use of aspirin for preventing metabolic, inflammatory, cardiovascular, and neoplastic diseases is limited by its potentially life-threatening side effects, especially gastrointestinal bleeding [40].

Recent studies indicated that ASA has a stimulatory effect on the osteogenic differentiation capacity of MSCs derived from various tissues and exerts protective effects on bone health by regulating the balance between bone formation and resorption [20,21,22,23,24]. Conversely, earlier research suggests that ASA exerts a contrary effect on adipogenesis of murine bone marrow MSCs (bmMSCs), tendon stem cells (TSCs), and endometrial stem cells (ESCs) [41,42,43]. However, to the best of our knowledge, the effect of ASA on adipogenesis of ASCs, the adipose tissue-native MSC population, is unknown. The present study investigates ASA’s impact on human ASCs in vitro, with a particular focus on adipogenic differentiation. In this regard, we hypothesize that ASA inhibits adipogenic differentiation of human ASCs in a dose-dependent manner. Additionally, we examine ASA’s effects on ASC morphology, migration, and clonogenic potential to comprehensively understand its influence on the cell source.

## 2. Results

### 2.1. Characterization of the Isolated Cell Population as ASCs

**Figure 1:**
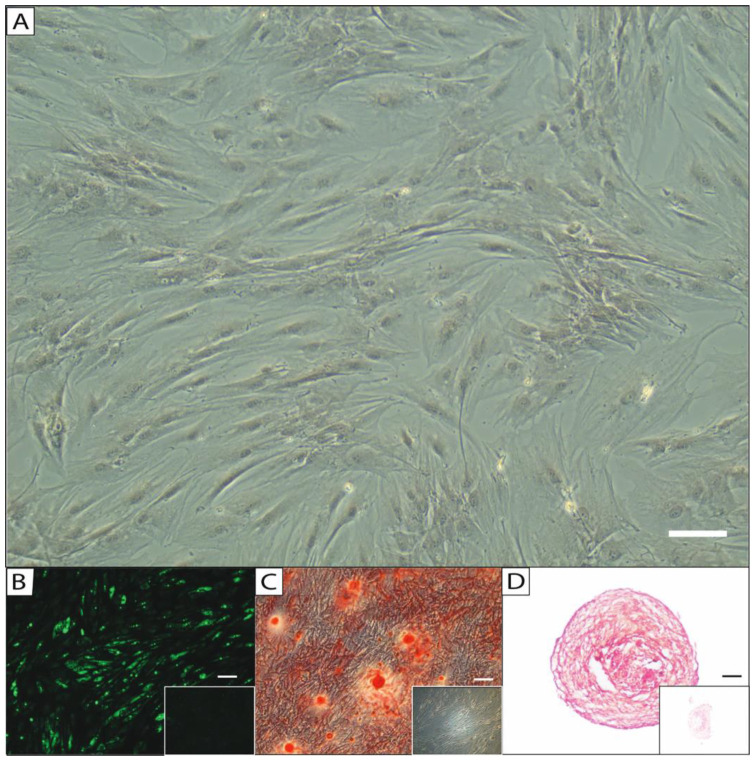
Morphology of isolated ASCs in passage 1 (**A**) with standard culture medium, after (**B**) adipogenic induction stained with BODIPY, (**C**) osteogenic induction stained with Alizarin S Red, and (**D**) chondrogenic differentiation stained with Safranin Orange. Unstimulated negative controls showed no formation of lipid vacuoles (**B**, insert), extracellular mineralization (**C**, insert), and proteoglycan deposition (**D**, insert). (10× magnification, scale bar = 200 μm.)

After isolation, the cells, used in passage 1, exhibited a spindle-shaped, fibroblast-like phenotype and showed adherence to plastic surfaces. Furthermore, their multipotency was demonstrated by visualizing trilineage differentiation (adipogenic, osteogenic, and chondrogenic) after induction with the corresponding media, using BODIPY, Alizarin S Red, and Safranin Orange staining, respectively (Figure 1). The isolated cell lines were previously characterized using flow cytometry as shown in Funke et al. [20], revealing a homogenous expression of positive (CD90, CD73, CD105, and CD44) and negative (CD45 and CD31) surface markers. As a result, in alignment with the joint statement from the International Federation for Adipose Therapeutics and Science (IFATS) and the International Society for Cellular Therapy (ISCT), these cells can be identified as ASCs [44].

### 2.2. ASA Concentrations ≤ 1000 µM Show No Significant Effect on ASC Migration Capacity

**Figure 2:**
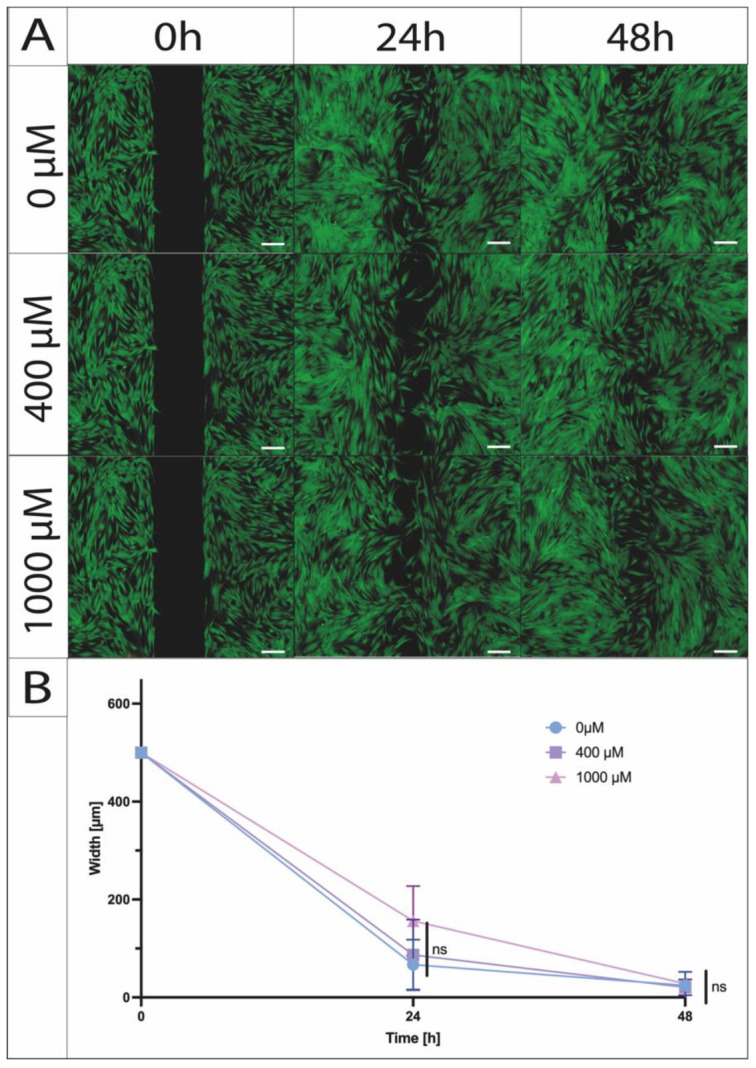
Migration potential of ASA-exposed ASCs (legend) using a migration test with a standardized slit width of 500 µm. (**A**) Visualized by live–dead staining after 24 and 48 h. (**B**) Quantification of gap width over time (n = 5; N = 3; ns = not significant; mean ± SD; and scale bar = 200 µm).

To visually display and quantify the migration of the ASCs, we conducted a standardized scratch assay using cell culture inserts with a gap width of 500 µm. The primary objective was to identify differences among the defined concentrations (400 µM and 1000 µM). Over 24 and 48 h, a consistent reduction in gap width was observed across concentrations of 400 µM and 1000 µM ASA, as well as the control (0 µM, undifferentiated). A statistical analysis revealed no significant differences between the evaluated ASA concentrations (Figure 2).

### 2.3. ASA Does Not Impair the Clonogenic Potential of ASCs

**Figure 3:**
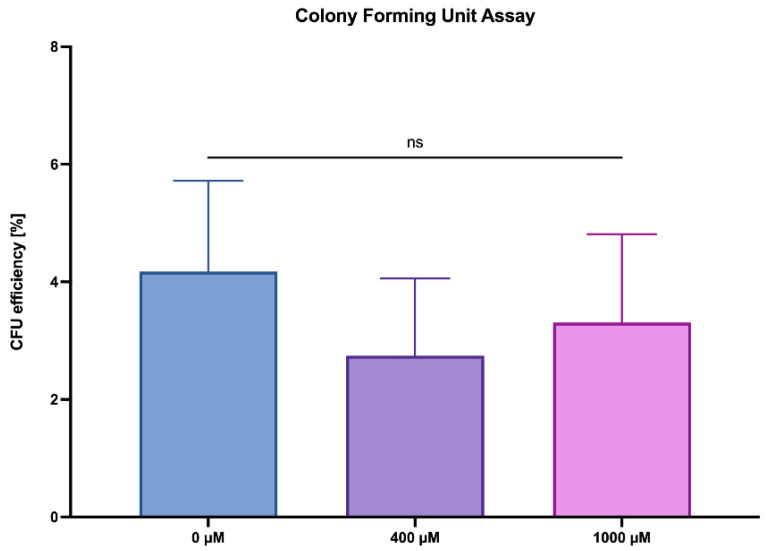
Clonogenic potential of ASCs under ASA-influence (x-axis) using a colony-forming unit assay visualized by Giemsa staining after 10 days of cultivation (n = 5; N = 3; ns = not significant; and mean ± SD).

A CFU-assay demonstrated the ability of self-renewal of the ASCs, with the control group (0 µM ASA, undifferentiated) forming an average of 25.1 colonies (±9.0 SD) and exhibiting a CFU efficiency of 4.2% (±1.3 SD). An additional analysis targeting the potential effects of concentrations of 400 µM and 1000 µM on clonogenic potential revealed no discernible differences, as indicated by the non-significant results. Specifically, 16.5 colonies (±8.6 SD) were observed upon exposure to 400 µM ASA, with a CFU efficiency of 2.74% (±1.43 SD). Meanwhile, exposure to 1000 µM resulted in 19.9 colonies (±8.6 SD) with a CFU efficiency of 3.31% (±1.43 SD) (Figure 3).

### 2.4. ASCs Show No Morphological Changes Under ASA Dose-Dependent Cultivation over 21 Days

**Figure 4:**
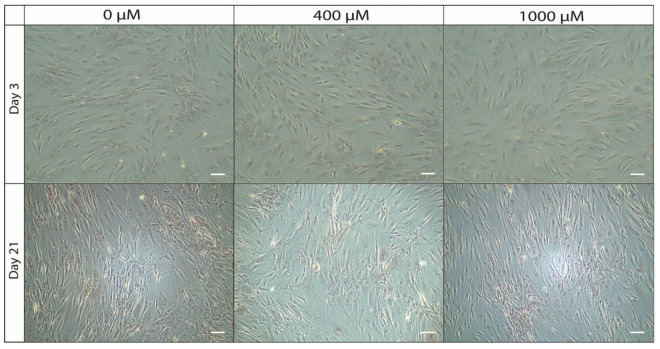
Morphology of ASCs after a 3- and 21-day cultivation period under the influence of 0 µM (control), 400 µM, and 1000 µM ASA (legend) at 10× magnification (scale bar = 200 µm).

Microscopic visualization of the two-dimensional cell cultures revealed no visible differences in cell morphology after 21 days when exposed to ASA. In the presence of ASA at concentrations of 400 µM and 1000 µM, the ASCs maintained their characteristic fibroblast-like appearance and demonstrated consistent confluence (exemplary illustration in Figure 4).

### 2.5. Morphological Changes During Adipogenic Differentiation of ASCs Under ASA Exposure

**Figure 5:**
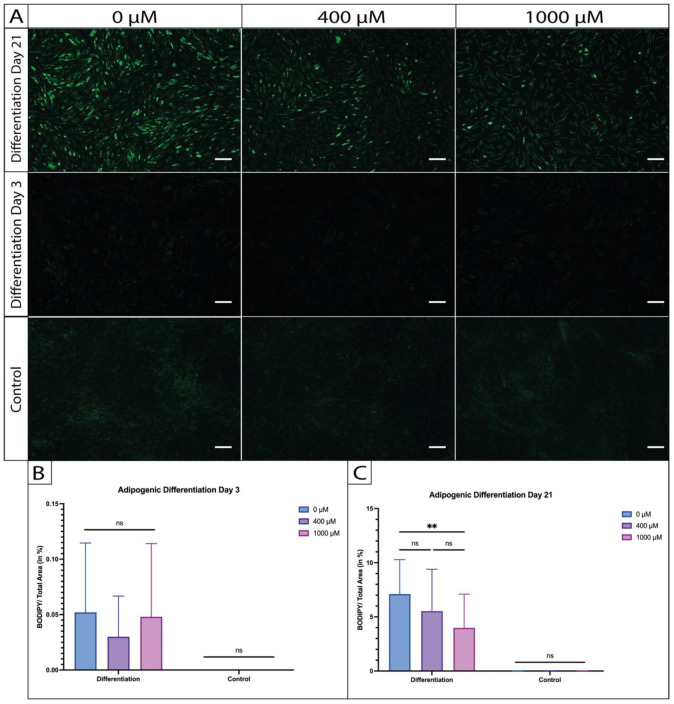
BODIPY staining of ASA-exposed and adipogenically differentiated ASCs captured at 5× magnification. (**A**) Comparison of the different time points (day 3 vs. day 21) of adipogenic differentiation under the influence of ASA (legend). (**B**) Quantified BODIPY-stained areas under ASA influence by day 3. (**C**) Quantified BODIPY-stained areas under ASA influence by day 21. (n = 5; N = 3; m = 6; ** *p* < 0.01; ns = not significant; mean ± SD; and scale bar = 200 µm.)

After inducing adipogenesis, the cells underwent BODIPY staining to visualize the developed intracellular lipid droplets following cultivation periods of 3 and 21 days. BODIPY staining conducted after a 3-day differentiation period showed no presence of lipid droplets (as shown in Figure 5A). However, by day 21, while the cells in all the differentiated groups, namely, the differentiated control group (0 µM ASA), 400 µM ASA, and 1000 µM ASA, appeared enlarged with lipid droplets in their cytoplasm, a closer analysis indicated inconsistencies in the lipid accumulation. Specifically, the differentiated control group (0 µM ASA) displayed more prominent lipid vacuoles compared to the aspirin-treated groups, with the most notable reduction observed in cells exposed to 1000 µM ASA (Figure 5A).

Quantification of the captured images confirmed the observed trend. On day 3, no significant differences were detected among the differentiated groups or when compared to their undifferentiated controls (0 µM vs. 400 µM vs. 1000 µM; ns; Figure 5B). By day 21, all the differentiated samples exhibited a significantly higher BODIPY ratio than their undifferentiated controls (0 µM: *p* = 0.0004; 400 µM: *p* = 0.0047; and 1000 µM: *p* = 0.0344). Additionally, the area occupied by BODIPY significantly decreased under 1000 µM ASA compared to the differentiated control without ASA treatment (*p* = 0.0025) as illustrated in Figure 5C. A trend, but no significance, could also be detected between 1000 µM and 400 µM ASA (ns), as well as 400 µM and the differentiated control (0 µM; ns).

### 2.6. ASA-Mediated Changes in Gene Expression During Adipogenic Differentiation of ASCs

**Figure 6:**
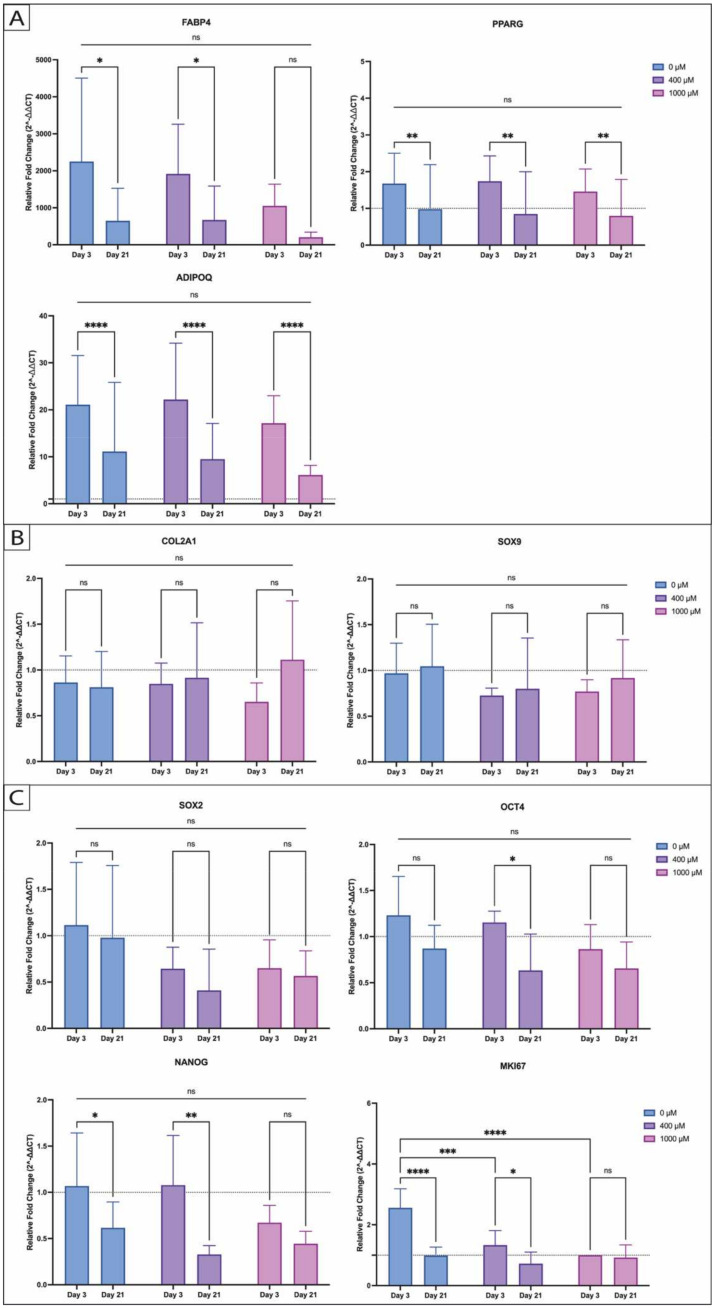
Genetic expression profile of (**A**) adipogenic differentiation (FABP4, PPARG, and ADIPOQ), (**B**) chondrogenic differentiation (COL2A1 and SOX9), (**C**) pluripotency markers (SOX2, OCT4, and NANOG), and proliferation marker MKI-67 under dose-dependent ASA exposure (legend) by day 3 and 21 measured by qPCR. Relative expressions were normalized to HPRT1 (housekeeping gene). (n = 5; N = 2; * *p* < 0.05; ** *p* < 0.01; *** *p* < 0.001; **** *p* < 0.0001; ns = not significant; and mean ± SD).

Finally, qPCR was performed to validate the observed changes at the gene expression level. The focus was on genes linked with adipogenesis, including *ADIPOQ*, *FABP4*, and *PPARG*, with the additional inclusion of chondrogenic differentiation markers *SOX9* and *COL2A1* to rule out any differentiation in this direction. Furthermore, the analysis integrated stem cell markers *SOX2*, *OCT4*, and *NANOG* and the proliferation marker MKI67 to provide comprehensive understanding of potential alterations in proliferation and stem cell characteristics. Our findings indicate a lesser expression of *FABP4* and *ADIPOQ* under the influence of 1000 µM ASA in comparison to the differentiated control group (0 µM ASA), though neither reached statistical significance (ns). Generally, the expression of adipogenic genes (*ADIPOQ*, *FABP4*, and *PPARG*) was consistently higher on day 3 compared to day 21. This temporal shift was observed for *ADIPOQ* and *PPARG* with equal significances across all three groups (0 µM vs. 400 µM vs. 1000 µM; *ADIPOQ*: *p* < 0.0001 each; and *PPARG*: *p* < 0.01 each), whereas *FABP4* was significantly reduced only in the control group (0 µM ASA, differentiated) and under 400 µM ASA (both *p* < 0.05) as shown in Figure 6A. The chondrogenic markers (Figure 6B) *SOX9* and *COL2A1* remained at basal levels after 3 and 21 days of adipogenic differentiation, regardless of ASA exposure. Furthermore, ASA concentrations did not seem to affect the expression of pluripotency-related genes (Figure 6C, including *SOX2*, *OCT4*, and *NANOG*). As adipogenesis progressed (day 21), a consistent downregulation of pluripotency markers was observed across all the groups, indicating an increasing commitment of the ASCs to the adipogenic lineage. The effects of ASA on the expression of the proliferation marker *MKi67* also appear to be noteworthy (Figure 6C, *MKi67*). The control group (0 µM ASA, differentiated) showed significantly higher expression on day 3 compared to the group under 400 µM ASA (*p* < 0.001) and 1000 µM ASA (*p* < 0.0001). By day 21, there were no longer any significant differences among the ASA-exposed samples. Nevertheless, a decrease over time was observed within the control group (0 µM ASA, differentiated; *p* < 0.0001) and the 400 µM induction group (*p* < 0.05) indicating growth arrest and progressed adipogenic differentiation.

## 3. Discussion

During the last decade, ASA further gained attention in stem cell research and regenerative medicine, where it has shown an influence on the regenerative properties of various stem cell sources, especially MSCs [20,21,22,23,41,43,45]. While the stimulatory effects of ASA on osteogenic differentiation have been well documented [20,21,22,23,46], its effect on adipogenic differentiation remains largely underexplored. This study is intended to evaluate the dose-dependent effects of ASA on the adipose tissue-native MSC population (ASCs) against the background of further unraveling ASAs role in ASC-adipogenesis and develop possible implications for cell therapy and regenerative medicine. A deeper understanding of these effects could enhance the precision of ASC therapies by promoting targeted differentiation and preventing the formation of unwanted cell types.

Building on prior research that evaluated osteogenesis, we focused on investigating ASA doses of 400 µM and 1000 µM [20,21,23,31,45]. These concentrations correspond to the typical clinical doses of ASA (325–3000 mg) used for analgesic and anti-inflammatory purposes, which result in blood plasma levels between 30 and 500 µM [47,48,49]. In an earlier study, we were able to show that doses between 100 µM and 1000 µM ASA did not significantly influence the viability and proliferation of ASCs in vitro. However, higher doses of ASA (10,000 µM and 16,000 µM) exhibited strong cytotoxic and anti-proliferative effects in ASCs and were therefore excluded from further experimentation, including this study [20]. To further unravel the influence of ASA on the ASC stem cell properties, we conducted a migration assay and CFU in the present study. The evaluation of the migration capacity of ASCs using a standardized scratch assay further revealed no influence of the examined ASA doses on ASC migration capacity, showing full viability and increasing cell confluency, continuously closing the slit width over the course of 48 h. Furthermore, cultivation under 400 µM and 1000 µM ASA marked no significant difference to the control group (0 µM ASA, undifferentiated) on the clonogenic potential of ASCs, as indicated by the CFU, which is regarded as the standard assay to demonstrate self-renewal potential [50]. Our results further revealed no visible changes in ASC morphology when cultivated over 21 days in a monolayer culture under ASA exposure, further underscoring the unlikelihood of any detrimental impact of ASA at these concentrations on ASC basic cellular functions over an extended culture period.

To address our hypothesis, we investigated the adipogenic differentiation potential of ASCs when exposed to 400 µM and 1000 µM ASA. To the best of our knowledge, this is the first study to evaluate the effect of ASA on the adipogenesis of human ASCs. Histological staining and quantification revealed a highly significantly reduced formation of lipid vacuoles after 21 days of stimulation with 1000 µM ASA compared to the control (0 µM ASA, differentiated). Although stimulation with 400 µM ASA also showed signs of impairment of ASC-adipogenesis in staining and quantification, this effect was not statistically significant. After 3 days of adipogenic differentiation, no significant differences were observed between the ASA-treated groups and the control. To confirm these observations at the genetic level, we performed a qPCR analysis. The focus of our investigations was mainly on the presentation of different adipogenic transcription factors and adipokines, whereby the most important inducing step of adipogenesis is mediated via PPARγ and its interaction with CEBP/a [19,51]. Under 1000 µM ASA, all three adipogenesis-related genes (*ADIPOQ*, *FABP4*, and *PPARG*) appeared to be less expressed in comparison to the control (0 µM ASA, differentiated) and the 400 µM group on both day 3 and day 21; however, these differences were not significant. After 21 days, we observed a reduction in the expression of ADIPOQ, FABP4, and PPARG that occurred independently of ASA treatment, relative to day 3. This pattern may reflect the attenuated importance of these genes in maintaining the mature adipocyte phenotype, highlighting their primary function in the early stages of differentiation. Remarkably, those changes were accompanied by a significantly lesser expression of the marker of proliferation MKI67 during early adipogenesis (day 3) under ASA exposure with increasing dosage. As discussed by Kajita et al., during early adipogenic stimulation, preadipocytes differentiate into small, replicating adipocytes, which lose their proliferative ability as they mature further in the adipogenesis process. Our results suggest that ASA interferes with this early stage of adipogenesis—characterized by a hybrid state of differentiation and proliferation—primarily by suppressing proliferation [52]. However, further experiments are necessary to confirm these observations.

Given the relatively underexplored impact of ASA on stem cell adipogenesis, there is only limited comparable data from other studies including stem cells and precursor cells. To summarize, Zhan et al. who evaluated the influence of ASA on the adipogenesis of bmMSCs over 21 days similarly found hat ASA decreased the contents of lipid droplets in a dose-dependent manner in 50, 100, 200, and 400 μM; the maximal decreased lipid accumulation was observed at a concentration of 400 μM ASA, while a concentration of 1000 µM was not evaluated [41]. Su et al. further found an ASA-induced inhibition of adipogenesis in mouse 3T3-L1 preadipocytes; this effect was also dose-dependent and more pronounced with increasing (0.01, 1, 10, 100, and 1000 μM ASA) concentrations [45]. In line with this, Wang et al. further reported an inhibition of adipogenesis in TSCs, as visualized by a dose-dependent reduction in Oil Red-positive lipid areas with increasing concentrations of ASA (250, 500, 1000, and 2000 μM ASA) after 14 days of adipogenic differentiation [43]. A similar inhibitory effect was found during adipogenic differentiation of human ESCs under stimulation with 2500 μM ASA [42]. All studies, including our own, consistently demonstrate that ASA inhibits adipogenesis across various stem cell types. This inhibitory effect appears to be dose-dependent, with higher concentrations of ASA leading to a more pronounced reduction in lipid accumulation. Notably, this effect is in stark contrast to the influence of ASA on osteogenic differentiation, where similar doses actually stimulate the differentiation process in ASCs, as well as in other types of MSCs [20,21,22,23]. These findings highlight the dual role of ASA, suggesting that while it impairs the formation of fat cells in an adipo-inductive environment, it concurrently promotes bone formation under osteo-inductive conditions. This further underscores the complex and dose-dependent nature of ASA’s influence on the reciprocal regulation between adipocyte and osteoblast differentiation in MSCs (as reviewed by Chen et al. [12]), which requires further investigation in the future.

To understand the possible points of attack of ASA in stem cell fate and adipogenesis, some theories have been examined in more detail so far. Su et al. demonstrated that ASA-induced inhibition of adipogenesis was p53-dependent and linked to the inactivation of the pentose phosphate pathway in murine preadipocytes [45]. In addition, Zhan et al. observed that the expression of the histone deacetylase HDAC9 increased in a dose-dependent manner when ASA was present during the adipogenic differentiation of bmMSCs. Their docking studies revealed a high affinity between HDAC9 and ASA, suggesting that HDAC9 may play a crucial role in the ASA-induced suppression of adipogenesis. [41]. In TSCs, ASA inhibited adipogenesis of the TSCs through inhibiting the PTEN/PI3K/AKT signaling pathway, which plays an important role in adipogenic differentiation and could be a new target for reversing adipogenesis and fatty acid formation [43,53,54]. Nevertheless, it remains unclear if those results also apply to ASCs and this circumstance requires additional investigation in the future. Furthermore, the current literature is inconclusive about the effect of COX-enzymes on adipogenesis, as both the promoting and inhibitory influences of COX-enzymes and the prostaglandins they produce have been described [55]. The extent to which ASA as an unselective COX-inhibitor enhances this balance remains the subject of research.

Beyond their role in adipose tissue development and homeostasis, numerous studies have highlighted that ASCs are pivotal in the development of obesity and related metabolic diseases. Previous research showed that a positive energy balance triggers the proliferation of ASCs, and when adipocytes reach their volume capacity, the newly formed ASCs are employed for de novo adipogenesis, further expanding the energy storage capacity of adipose tissue [56,57]. In addition, the dysfunction of adipocytes in obesity can affect both the number and function of ASCs, leading to irregular adipose tissue remodeling (adiposopathy), which is associated with a heightened risk of metabolic disorders [57,58]. In this context, it would be tempting to assume that ASA might be a potential therapeutic agent in the treatment of obesity and metabolic syndrome by inhibition of ASC-adipogenesis. Indeed, a previous study indicated that low-dose aspirin alleviates hyperlipidemia by reducing the transcript levels of PPARγ, the master regulator of adipogenesis. In addition to its anti-adipogenic effects, ASA also reduces the obesity-induced expression of inflammatory cytokines (IL-6, TNFα, and MCP-1) and adhesion molecules (ICAM-I and VCAM-I) while enhancing insulin sensitivity in high-fat diet-induced obese mice [39]. However, obesity is a complex interplay of genetic, environmental, and behavioral factors, which means that while ASA’s effects on adipogenesis and inflammation are promising, they may represent only one aspect of a multifaceted approach to treatment. Therefore, further studies are necessary to fully understand the mechanisms by which ASA can be integrated into broader therapeutic strategies in treatment of adiposopathy and metabolic disease.

In the context of ASCs’ application in cell therapies and regenerative medicine, such as tissue engineering, the inhibitory effect of ASA on ASC-adipogenesis warrants careful consideration. In plastic and reconstructive surgery, for example, fat graft enrichment with ASCs or the SVF is a well-established technique to enhance graft survival rates in autologous fat grafting (cell-assisted lipotransfer) used for soft tissue augmentation [59]. ASCs play a crucial role in adipose tissue remodeling after cell-assisted lipotransfer by contributing to angiogenesis through differentiation into endothelial cells and undergoing adipogenesis, which effectively improves fat graft retention [60]. Moreover, ASA’s well-known anti-inflammatory properties could have implications for regenerative medicine by potentially modulating the inflammatory response in conditions where inflammation negatively impacts ASC function, such as during wound healing or tissue repair. In this clinical context, the use of ASA should be carefully evaluated and ideally avoided to not endanger the success of the fat graft. Additionally, further research is necessary to determine whether other COX-inhibitors, particularly those commonly used for post-operative pain management (such as Ibuprofen and Naproxen), exhibit a similar inhibitory effect on adipogenesis.

While this study provides valuable insights into the effects of ASA on adipogenic differentiation of ASCs, several limitations must be acknowledged. Firstly, this study’s limited sample size (n = 5) and homogeneous stem cell donor population, consisting exclusively of Caucasian females and focusing on subcutaneous thigh adipose tissue, may constrain the robustness of the findings and their applicability to broader demographics and diverse adipose tissue types. Additionally, the doses of ASA used in this study, while reflective of typical clinical ranges, might not fully capture the complexity of ASA’s effects at different concentrations or in varying physiological contexts, particularly with regard to drug metabolism and distribution. Moreover, this study did not include experiments to confirm the observed trends in gene expression at the protein level, and since it was conducted in vitro, the results should be interpreted with caution regarding their implications and applicability in a clinical setting. These limitations highlight the need for further research involving larger and more diverse sample sizes, as well as animal models, to identify relevant molecular pathways and gain a more comprehensive understanding of ASA’s impact on stem cell differentiation and its potential implications for regenerative medicine.

## 4. Materials and Methods

### 4.1. Donor Demographics and Ethics Statement

For this study, the adipose tissue samples were obtained during elective body-contouring procedures at the Department of Plastic Surgery, LMU Munich University Hospital. The surgical interventions were performed between April and May 2022 using waterjet-assisted liposuction technology (Body Jet evo, Human Med AG, Schwerin, Germany). The study population comprised five female Caucasian individuals with thigh-region lipoaspirates. The participant demographics showed a mean age of 43.4 ± 13.3 years and mean body mass index (BMI) of 35.5 ± 3.21 kg/m². Prior to surgery, all the donors provided written consent for participation in this study, having tested negative for HIV, hepatitis B, and hepatitis C. Additionally, the donors were non-smokers, reported no significant pre-existing medical conditions, and stated they did not use aspirin regularly or sporadically. This study was approved by the local ethics committee under registration number 22-0502 and was conducted in accordance with the Declaration of Helsinki.

### 4.2. Cell Isolation, Cultivation, and Characterization

For the enzymatic isolation of ASCs, the procedure described by Funke et al. was followed using a collagen type II solution (Worthington Biochemical Corp., Lakewood, NJ, USA; Ref. LS004176) [20]. The isolated cells were incubated under humidified conditions (21% O_2_, 5% CO_2_, 37 °C; Heracell Vios 160i, Thermo Fisher Scientific, Waltham, MA, USA, serial number: 42231008) in standard culture medium (88% Dulbecco’s modified eagle medium (DMEM, Gibco^TM^, Thermo Fisher Scientific, Waltham, MA, USA, Ref: 11956-092), 10% fetal bovine serum (FBS, Gibco^TM^, Thermo Fisher Scientific, Paisley Scotland, Ref: 10270-106), 1% penicillin 10,000 U/mL/streptomycin 10 mg/mL (PAN-Biotech, Aidenbach, Germany; LOT: 4260422), and 1% amphotericin B 250 µg/mL (Sigma-Aldrich, St. Louis, MO, USA, Ref: A2942)). Media changes were performed every 3–4 days until 80% confluency was achieved. For long-term storage, the ASCs were cryopreserved in liquid nitrogen with a cryomedium consisting of 10% dimethyl sulfoxide (DMSO, Carl-Roth, Karlsruhe, Germany, Ref: 4720.2) and 90% FBS. Unless otherwise specified, all the experiments were conducted using passage 1 cells from five different donors (n = 5) with three biological replicates per donor (N = 3 per donor). For the visualization of the cell morphology, images were captured using the Axio Observer KMAT (Zeiss, Oberkochen, Germany) at 10× magnification after 21 days. In addition to adipogenic differentiation (see below), osteogenic and chondrogenic differentiation were conducted as described previously to confirm the multilineage differentiation potential of the isolated cell population [61,62]. Therefore, histological staining with Alizarin S Red (Sigma-Aldrich, St. Louis, MO, USA, ECM815, PartNo. 2003999) was performed to highlight calcium deposits as a sign of osteogenesis [20,61] and Safranin Orange (Sigma-Aldrich, St. Louis, MO, USA, S2255) staining for the detection of proteoglycan present in cartilage [62].

### 4.3. Composition and Dilution of ASA Stock Solution

To prepare the ASA stock solution, ASA (>99.0%, Sigma-Aldrich, St. Louis, MO, USA, Ref: A5376) was weighed under sterile conditions and dissolved in standard culture medium at a concentration of 3 mg/mL (16,651 µM) at 25 °C, following the manufacturer’s instructions. Based on the literature and our previous findings, the final experiments were conducted using ASA at concentrations of 0 µM (control), 400 µM, and 1000 µM, which were prepared by serial dilution of the stock solution [20,21,41].

### 4.4. Cell Migration Assay

To visually track cell migration influenced by the different ASA concentrations, a migration assay was performed with fluorescence imaging after 0 h, 24 h, and 48 h. The ASCs were seeded into Ibidi Cell Culture Inserts (Ibidi GmbH, Martinsried, Germany) at a concentration of 10,000 cells/70 µL per chamber. According to the manufacturer’s specification, the gap width of the silicone chambers is 500 µm. The seeding and cultivation adhered to the manufacturer’s protocol [63]. After overnight incubation with culture medium, the silicone chambers were carefully removed, and the samples were further incubated with culture and ASA medium. The control measurements were performed immediately after chamber removal, followed by imaging of the remaining samples after 24 h and 48 h. For visualization, the cells were stained using a fluorescent LIVE–DEAD staining (fluorescein diacetate (FDA, Sigma-Aldrich, St. Louis, MO, USA, Ref: F7378-25G) and propidium iodide (PI, Sigma-Aldrich, St. Louis, MO, USA, Ref: P4864)) as previously described by Funke et al. [20]. The images were acquired using the Axio Observer KMAT microscope (Zeiss, Oberkochen, Germany) at 5× magnification, capturing data in the 526 nm (LDGre) and 613 nm (LDRed) channels. The quantitative assessment of the gap width was evaluated with the Wound Healing Size Tool, a plugin for the open-source software ImageJ 1.53 t for Mac (https://imagej.net/ij/, accessed on 20 February 2024) [64].

### 4.5. Colony-Forming Unit Assay

For the colony-forming unit (CFU) assay, the ASCs were plated at a density of 10 cells/cm^2^ per P100 petri dish (100/20 mm, Greiner Bio-One, Austria, Ref: 664160) and cultured in 10 mL medium (ASA concentrations: 0 µM, 400 µM, and 1000 µM) over 10 days. Afterwards, the plates were fixed with methanol (Methanol 99,9%, Fisher Chemical, Thermo Fisher Scientific, Waltham, MA, USA, Ref: M/4000/PC17) and stained with Giemsa Working Solution (Sigma-Aldrich, St. Louis, MO, USA, Ref: 32884-250 mL). Colonies with >8 cells were counted under the microscope and the CFU efficiency was then calculated using the following formula: CFU [%] = (number of colonies/number of cells plated) × 100.

### 4.6. Induction and Quantification of Adipogenic Differentiation of ASCs

To investigate ASA-influenced adipogenic differentiation, BODIPY staining targeting lipid droplets was carried out. The ASCs were seeded in six-well plates at a density of 5 × 10^4^ cells per well. After a five-day incubation in standard medium, ASA exposure (0 µM, 400 µM, and 1000 µM) as well as the treatment with differentiation medium started over a period of either 3 or 21 days. The adipogenic differentiation medium consisted of 98% adipogenic differentiation medium (StemMACs AdipoDiff Media, Miltenyi Biotec, Bergisch Gladbach, Germany, Ref: 130-091-677), 1% penicillin/streptomycin, and 1% amphotericin B. For histological staining with the fluorescent dye BODIPY, the cells were rinsed with Dulbecco’s Phosphate-Buffered Saline DPBS (Gibco^TM^, Thermo Fisher Scientific, Paisley, Scotland; Ref: 14190-094) and fixed with 4% formaldehyde (Microcos GmbH, Garching, Germany, LOT: 100816) for 30 min at room temperature. After three washing steps with DPBS, 2 mL BODIPY working solution (0.4 µL BODIPY stock solution, consisting of 10 mg/mL BODIPY 493/503 staining solution (Invitrogen, Thermo Fisher Scientific, Waltham, MA, USA, D3922) in absolute ethanol (Merck, Sigma-Aldrich, St. Louis, MO, USA, Ref: 1009861000), in 1 mL DPBS) was added per well and incubated at 37 °C for 15 min. Following another washing step with DPBS, the cells were imaged using an Axio Observer KMAT fluorescence microscope (Zeiss, Oberkochen, Germany) at 5× magnification with a GFP fluorescence filter. For each well, six standardized images were taken to ensure consistent analysis across the samples (m = 6). The quantification of the adipogenically differentiated cells was performed using the open-source software ImageJ 1.53 t for Mac (https://imagej.net/ij/, accessed on 6 July 2024) with the macro “measure stack” as described by Wiggenhauser et al. [65]. The software analyzed the proportion of colored areas to the total number of pixels, providing a relative expression of areas covered in lipid droplets. The area was measured at a uniformly defined threshold value (20–255) and calculated as the percentage (%) of the total cell-occupied area.

### 4.7. RNA Purification, Reverse Transcription into cDNA, and Quantitative PCR Analysis

The cell seeding and culture conditions for the quantitative polymerase chain reaction (qPCR) were consistent with those used during adipogenic differentiation, with a reduction from biological triplicates to duplicates (n = 5; N = 2). RNA extraction was carried out using the RNeasy mini kit (Qiagen, Hilden, Germany, Ref: 74104) following the manufacturer’s instructions. Prior to purification, cell pellets from the samples were stored at −80 °C with 50 µL each of “RNAlater” RNA stabilization reagent (Qiagen, Germany, LotNo: 160027967). The RNA quantities were measured using the Infinite M Plex plate reader and i-controlTM software (both Tecan Trading AG, Männedorf, Switzerland) at a wavelength of 260 nm (ratio 280 nm).

The transcriptor first-strand cDNA synthesis kit (Hoffmann-La Roche, Basel, Switzerland, Ref: 04897030001) was then applied for cDNA synthesis. After preparation of the primer mix (2 µL PCR grade water, 2 µL random hexamer primer, and 1 µL oligo (dT)_18_ primer) and the reverse transcription mix (4 µL Transcriptor RT Reaction Buffer, 0.5 µL Protector RNase Inhibitor, 2 µL Deoxynucleotide Mix 1 mM, and 0.5 µL Transcriptor Reverse Transcriptase), 5 µL of the primer mixes was dispensed into the respective PCR reaction tubes (PCR SingleCap 8-piece-SoftStrips 0.2 mL, Biozym Scientific, Hessisch Oldendorf, Germany, LotNo. 21072). Next, 10 µL of the pre-expressed RNA samples was pipetted into triplicates and the suspension was denatured for 10 min at +65 °C in a thermocycler (Biometra TOne, Analytik-Jena, Jena, Germany). After cooling for 20 min at +4 °C, 7 µL of the reverse transcription mix was added and the samples were incubated in the thermocycler according to the following program: 10 min at 25 °C, 60 min at 50 °C, 5 min at 85 °C, and 30 min at 4 °C. The samples were stored at −20 °C until further processing.

As part of the subsequent quantitative polymerase chain reaction (qPCR), 5 µL of each of the corresponding cDNA samples was pipetted onto a PCR plate (VWR^®^ PCR Plate, 96 Well, ABI type, VWR, Avantor, Radnor, PA, USA, Ref: 211-0317) and mixed with 5 µL of a master mix consisting of 10 µL InnuMix qPCR MasterMix sample (Analytik Jena, Jena, Germany, Ref: 845-AS-1201000), 1 µL forward primer, 1 µL reverse primer, and 3 µL DEPC-treated, DNase- and RNase-free water (Carl Roth, Karlsruhe, Germany, CasNo. 7732-18-5). An overview of the primers used can be found in Table 1. The prepared samples were then subjected to a qPCR run on the qTower3 G touch, beginning with an initial denaturation step at 95 °C for 2 min. This was followed by 40 cycles of amplification, each consisting of three phases: denaturation at 95 °C for 30 s, annealing at 60 °C for 1 min, and a subsequent scanning phase at 68 °C for 30 s. These carefully controlled conditions ensured the precise amplification and detection of the target DNA sequences.

The gene expression levels were quantified using the ∆∆Ct method and the following formula: Fold gene expression = 2^−(∆∆Ct)^. Initially, the CT values, representing the PCR cycles for a constant fluorescence level, were normalized against the housekeeping gene (HKG) HPRT1 (ΔCt = Ct_target gene_ − ΔCt_HKG_). Then, the average ΔCt of the non-differentiated control group was subtracted from the ΔCt values of the differentiated samples to determine the ∆∆Ct value (∆∆Ct = ∆Ct_ASA_treatment_ − ∆Ct_control_). This calculation was performed separately for each treatment condition (0 µM, 400 µM, and 1000 µM).

### 4.8. Statistical Evaluation and Data Illustration

The statistical evaluations were performed using GraphPad Prism v10 for Mac (GraphPad Software, San Diego, CA, USA), while the illustrations were generated using Adobe Illustrator 2023 (Adobe Creative Cloud, Adobe Inc., San Jose, CA, USA). A Shapiro–Wilk test was conducted to assess the data normality, and depending on the outcome, either an unpaired *t*-test (Student’s *t*-test) or a Mann–Whitney U-test was applied for statistical analysis. For multiple group comparisons, a two-way analysis of variance (ANOVA) was used, followed by Bonferroni’s post hoc multiple comparison test if significant differences were found. The data are expressed as the mean ± standard deviation (SD), and statistical significance was considered for *p*-values < 0.05.

## 5. Conclusions

In conclusion, this research highlights the dose-dependent effects of ASA on the adipogenic differentiation of ASCs, demonstrating that ASA significantly inhibits lipid accumulation at higher concentrations (1000 µM) while not affecting the migration capacity, clonogenic potential, and morphology. Although lower-dose ASA treatment (400 µM) also showed signs of impaired adipogenesis, these effects were not statistically significant. This contrasts with ASA’s established role in promoting osteogenic differentiation, highlighting its complex and context-specific actions on ASCs. These findings suggest that ASA influences ASC-adipogenesis in a dose-dependent manner, with potential implications for regenerative medicine and metabolic disease treatment. Future research is needed to further elucidate the mechanisms underlying ASA’s effects on stem cell regulation and explore its broader therapeutic potential.

## Figures and Tables

**Table 1:** Primer sequences and probe design for qPCR.

**Gen**	**Full Name**	**Forward Primer** **Sequence (5′→3′)**	**Reverse Primer** **Sequence (5′→3′)**	**Length**	**NCBI RefSeq**
MKi67	Marker of proliferation Ki-67	AATCACTAAAATGCCCTGCC	CTTCTTTCACACCTACTTTCCC	11,636 bp	NM_001145966
PPARG	Peroxisome proliferator-activated receptor gamma	AAGAAGCCAACACTAAACCAC	TTACGGAGAGATCCACGGAG	1323 bp	NM_001330615
HPRT1	Hypoxanthine phosphoribosyltransferase 1	AGATGGTCAAGGTCGCAAG	AAGGGCATATCCTACAACAAAC	1395 bp	NM_000194
FABP4	Fatty acid binding protein 4	CCAGGAATTTGACGAAGTCAC	CACCACCAGTTTATCATCCTC	911 bp	NM_001442.3
OCT4/POU5F1	POU domain, class 5, transcription factor 1 isoform 2	AAAGAGAAAGCGAACCAGTATC	TACAGAACCACACTCGGAC	1579 bp	NP_001167002.1
ADIPOQ	Adiponectin	GTAAATTCCACTGCAACATTCC	TGAAGAGCATAGCCTTGTCC	4593 bp	NM_001177800.2
NANOG	Nanog homebox	TCTCTCCTCTTCCTTCCTCC	AGTTCTGGTCTTCTGTTTCTTG	1395 bp	NM_024865.4
COL2A1	Collagen type II alpha 1 chain	TCCATTCATCCCACCCTCTC	AGTTTCCTGCCTCTGCCTTG	5059 bp	NM_001844.5
SOX2	SRY-box transcription factor 2	GCTCGCAGACCTACATGAAC	GGAGGAAGAGGTAACCACAG	2512 bp	NM_003106
SOX9	SRY-box transcription factor 9	AGTTTCTTTGTATTCCTCACCC	TCAAAACACACACACACCC	3931 bp	NM_000346.4

## Data Availability

The authors declare that the data supporting the findings of this study are available within this paper. Should any raw data files be needed, they are available from the corresponding author upon reasonable request.

## References

[1] Huang S.J., Fu R.H., Shyu W.C., Liu S.P., Jong G.P., Chiu Y.W., Wu H.S., Tsou Y.A., Cheng C.W., Lin S.Z. (2013). Adipose-derived stem cells: Isolation, characterization, and differentiation potential. Cell Transpl..

[2] Hildreth A.D., Ma F., Wong Y.Y., Sun R., Pellegrini M., O’Sullivan T.E. (2021). Single-cell sequencing of human white adipose tissue identifies new cell states in health and obesity. Nat. Immunol..

[3] Palumbo P., Lombardi F., Siragusa G., Cifone M.G., Cinque B., Giuliani M. (2018). Methods of Isolation, Characterization and Expansion of Human Adipose-Derived Stem Cells (ASCs): An Overview. Int. J. Mol. Sci..

[4] Zuk P.A., Zhu M., Ashjian P., De Ugarte D.A., Huang J.I., Mizuno H., Alfonso Z.C., Fraser J.K., Benhaim P., Hedrick M.H. (2002). Human adipose tissue is a source of multipotent stem cells. Mol. Biol. Cell.

[5] Zuk P.A., Zhu M., Mizuno H., Huang J., Futrell J.W., Katz A.J., Benhaim P., Lorenz H.P., Hedrick M.H. (2001). Multilineage cells from human adipose tissue: Implications for cell-based therapies. Tissue Eng..

[6] Strem B.M., Hicok K.C., Zhu M., Wulur I., Alfonso Z., Schreiber R.E., Fraser J.K., Hedrick M.H. (2005). Multipotential differentiation of adipose tissue-derived stem cells. Keio J. Med..

[7] Van Dongen J.A., Harmsen M.C., van der Lei B., Stevens H.P. (2018). Augmentation of Dermal Wound Healing by Adipose Tissue-Derived Stromal Cells (ASC). Bioengineering.

[8] Torres-Torrillas M., Rubio M., Damia E., Cuervo B., Del Romero A., Pelaez P., Chicharro D., Miguel L., Sopena J.J. (2019). Adipose-Derived Mesenchymal Stem Cells: A Promising Tool in the Treatment of Musculoskeletal Diseases. Int. J. Mol. Sci..

[9] Milan G., Conci S., Sanna M., Favaretto F., Bettini S., Vettor R. (2021). ASCs and their role in obesity and metabolic diseases. Trends Endocrinol. Metab..

[10] Cheung Y.M., Chook C.Y., Yeung H.W., Leung F.P., Wong W.T. (2023). A Wrong Fate Decision in Adipose Stem Cells upon Obesity. Cells.

[11] Zhang Y., Khan D., Delling J., Tobiasch E. (2012). Mechanisms underlying the osteo- and adipo-differentiation of human mesenchymal stem cells. Sci. World J..

[12] Chen Q., Shou P., Zheng C., Jiang M., Cao G., Yang Q., Cao J., Xie N., Velletri T., Zhang X. (2016). Fate decision of mesenchymal stem cells: Adipocytes or osteoblasts?. Cell Death Differ..

[13] Ambele M.A., Dhanraj P., Giles R., Pepper M.S. (2020). Adipogenesis: A Complex Interplay of Multiple Molecular Determinants and Pathways. Int. J. Mol. Sci..

[14] Li H.X., Luo X., Liu R.X., Yang Y.J., Yang G.S. (2008). Roles of Wnt/beta-catenin signaling in adipogenic differentiation potential of adipose-derived mesenchymal stem cells. Mol. Cell Endocrinol..

[15] Sandel D.A., Liu M., Ogbonnaya N., Newman J.J. (2018). Notch3 is involved in adipogenesis of human adipose-derived stromal/stem cells. Biochimie.

[16] Fontaine C., Cousin W., Plaisant M., Dani C., Peraldi P. (2008). Hedgehog signaling alters adipocyte maturation of human mesenchymal stem cells. Stem Cells.

[17] Cristancho A.G., Lazar M.A. (2011). Forming functional fat: A growing understanding of adipocyte differentiation. Nat. Rev. Mol. Cell Biol..

[18] Bennett C.N., Ross S.E., Longo K.A., Bajnok L., Hemati N., Johnson K.W., Harrison S.D., MacDougald O.A. (2002). Regulation of Wnt signaling during adipogenesis. J. Biol. Chem..

[19] Wu Z., Rosen E.D., Brun R., Hauser S., Adelmant G., Troy A.E., McKeon C., Darlington G.J., Spiegelman B.M. (1999). Cross-regulation of C/EBP alpha and PPAR gamma controls the transcriptional pathway of adipogenesis and insulin sensitivity. Mol. Cell.

[20] Funke S., Wiggenhauser P.S., Grundmeier A., Taha S., Fuchs B., Birt A., Koban K., Giunta R.E., Kuhlmann C. (2024). Aspirin Stimulates the Osteogenic Differentiation of Human Adipose Tissue-Derived Stem Cells In Vitro. Int. J. Mol. Sci..

[21] Li Y., Luo Z., Xu X., Li Y., Zhang S., Zhou P., Sui Y., Wu M., Luo E., Wei S. (2017). Aspirin enhances the osteogenic and anti-inflammatory effects of human mesenchymal stem cells on osteogenic BFP-1 peptide-decorated substrates. J. Mater. Chem. B.

[22] Abd Rahman F. (2021). Gene expression profiling on effect of aspirin on osteogenic differentiation of periodontal ligament stem cells. BDJ Open.

[23] Vukovic M., Lazarevic M., Mitic D., Jaksic Karisik M., Ilic B., Andric M., Jevtic B., Roganovic J., Milasin J. (2022). Acetylsalicylic-acid (ASA) regulation of osteo/odontogenic differentiation and proliferation of human dental pulp stem cells (DPSCs) in vitro. Arch. Oral. Biol..

[24] Xie Y., Pan M., Gao Y., Zhang L., Ge W., Tang P. (2019). Dose-dependent roles of aspirin and other non-steroidal anti-inflammatory drugs in abnormal bone remodeling and skeletal regeneration. Cell Biosci..

[25] Mahdi J.G., Mahdi A.J., Mahdi A.J., Bowen I.D. (2006). The historical analysis of aspirin discovery, its relation to the willow tree and antiproliferative and anticancer potential. Cell Prolif..

[26] Montinari M.R., Minelli S., De Caterina R. (2019). The first 3500 years of aspirin history from its roots—A concise summary. Vasc. Pharmacol.

[27] Bunting S., Moncada S., Vane J.R. (1983). The prostacyclin--thromboxane A2 balance: Pathophysiological and therapeutic implications. Br. Med. Bull..

[28] Vane J.R. (1971). Inhibition of prostaglandin synthesis as a mechanism of action for aspirin-like drugs. Nat. New Biol..

[29] Toth L., Muszbek L., Komaromi I. (2013). Mechanism of the irreversible inhibition of human cyclooxygenase-1 by aspirin as predicted by QM/MM calculations. J. Mol. Graph. Model..

[30] Vane J.R., Botting R.M. (2003). The mechanism of action of aspirin. Thromb. Res..

[31] Vane J.R., Botting R.M. (1996). Mechanism of action of anti-inflammatory drugs. Scand. J. Rheumatol. Suppl..

[32] Rezabakhsh A., Mahmoodpoor A., Soleimanpour M., Shahsavarinia K., Soleimanpour H. (2021). Clinical Applications of Aspirin as a Multi-potent Drug Beyond Cardiovascular Implications: A Proof of Concept for Anesthesiologists—A Narrative Review. Anesth. Pain Med..

[33] Tsoi K.K.F., Ho J.M.W., Chan F.C.H., Sung J.J.Y. (2019). Long-term use of low-dose aspirin for cancer prevention: A 10-year population cohort study in Hong Kong. Int. J. Cancer.

[34] Bibbins-Domingo K., Force U.S.P.S.T. (2016). Aspirin Use for the Primary Prevention of Cardiovascular Disease and Colorectal Cancer: U.S. Preventive Services Task Force Recommendation Statement. Ann. Intern. Med..

[35] Kwon S., Ma W., Drew D.A., Klempner S.J., Leonardo B.M., Flynn J.J., Cao Y., Giovannucci E.L., Bao Y., Fuchs C.S. (2022). Association Between Aspirin Use and Gastric Adenocarcinoma: A Prospective Cohort Study. Cancer Prev. Res..

[36] Chan A.T., Giovannucci E.L., Meyerhardt J.A., Schernhammer E.S., Curhan G.C., Fuchs C.S. (2005). Long-term use of aspirin and nonsteroidal anti-inflammatory drugs and risk of colorectal cancer. JAMA.

[37] Daly P.A., Krieger D.R., Dulloo A.G., Young J.B., Landsberg L. (1993). Ephedrine, caffeine and aspirin: Safety and efficacy for treatment of human obesity. Int. J. Obes. Relat. Metab. Disord..

[38] Kim J.K., Kim Y.J., Fillmore J.J., Chen Y., Moore I., Lee J., Yuan M., Li Z.W., Karin M., Perret P. (2001). Prevention of fat-induced insulin resistance by salicylate. J. Clin. Investig..

[39] Roy S., Bhowmik D.R., Begum R., Amin M.T., Islam M.A., Ahmed F., Hossain M.S. (2022). Aspirin attenuates the expression of adhesion molecules, risk of obesity, and adipose tissue inflammation in high-fat diet-induced obese mice. Prostaglandins Other Lipid Mediat..

[40] Huang E.S., Strate L.L., Ho W.W., Lee S.S., Chan A.T. (2011). Long-term use of aspirin and the risk of gastrointestinal bleeding. Am. J. Med..

[41] Zhan Y., He Z., Liu X., Miao N., Lin F., Xu W., Mu S., Mu H., Yuan M., Cao X. (2018). Aspirin-induced attenuation of adipogenic differentiation of bone marrow mesenchymal stem cells is accompanied by the disturbed epigenetic modification. Int. J. Biochem. Cell Biol..

[42] Park S.R., Kim S.R., Min E.K., Oh B.C., Jung Y., Kim Y.H., Lee H.Y. (2023). Unveiling the potential effects of acetylsalicylic acid: Insights into regeneration in endometrial stem cells. Cell Commun. Signal.

[43] Wang Y., He G., Wang F., Zhang C., Ge Z., Zheng X., Deng H., Yuan C., Zhou B., Tao X. (2019). Aspirin inhibits adipogenesis of tendon stem cells and lipids accumulation in rat injury tendon through regulating PTEN/PI3K/AKT signalling. J. Cell Mol. Med..

[44] Bourin P., Bunnell B.A., Casteilla L., Dominici M., Katz A.J., March K.L., Redl H., Rubin J.P., Yoshimura K., Gimble J.M. (2013). Stromal cells from the adipose tissue-derived stromal vascular fraction and culture expanded adipose tissue-derived stromal/stem cells: A joint statement of the International Federation for Adipose Therapeutics and Science (IFATS) and the International Society for Cellular Therapy (ISCT). Cytotherapy.

[45] Su Y.F., Yang S.H., Lee Y.H., Wu B.C., Huang S.C., Liu C.M., Chen S.L., Pan Y.F., Chou S.S., Chou M.Y. (2014). Aspirin-induced inhibition of adipogenesis was p53-dependent and associated with inactivation of pentose phosphate pathway. Eur. J. Pharmacol..

[46] Zhang Y., Ding N., Zhang T., Sun Q., Han B., Yu T. (2019). A Tetra-PEG Hydrogel Based Aspirin Sustained Release System Exerts Beneficial Effects on Periodontal Ligament Stem Cells Mediated Bone Regeneration. Front. Chem..

[47] Alfonso L., Ai G., Spitale R.C., Bhat G.J. (2014). Molecular targets of aspirin and cancer prevention. Br. J. Cancer.

[48] Dovizio M., Bruno A., Tacconelli S., Patrignani P. (2013). Mode of action of aspirin as a chemopreventive agent. Recent Results Cancer Res..

[49] Drummond A.H., MacIntyre D.E., Olverman H.J. (1977). Aspirin at therapeutic concentrations does not affect 5-hydroxytryptamine uptake by platelets. Br. J. Pharmacol..

[50] Alberton P., Popov C., Pragert M., Kohler J., Shukunami C., Schieker M., Docheva D. (2012). Conversion of human bone marrow-derived mesenchymal stem cells into tendon progenitor cells by ectopic expression of scleraxis. Stem Cells Dev..

[51] Lee J.E., Ge K. (2014). Transcriptional and epigenetic regulation of PPARgamma expression during adipogenesis. Cell Biosci..

[52] Kajita K., Mori I., Kitada Y., Taguchi K., Kajita T., Hanamoto T., Ikeda T., Fujioka K., Yamauchi M., Okada H. (2013). Small proliferative adipocytes: Identification of proliferative cells expressing adipocyte markers. Endocr. J..

[53] Wang Y., Liu Y., Fan Z., Liu D., Wang F., Zhou Y. (2017). IGFBP2 enhances adipogenic differentiation potentials of mesenchymal stem cells from Wharton’s jelly of the umbilical cord via JNK and Akt signaling pathways. PLoS ONE.

[54] Song F., Jiang D., Wang T., Wang Y., Lou Y., Zhang Y., Ma H., Kang Y. (2017). Mechanical Stress Regulates Osteogenesis and Adipogenesis of Rat Mesenchymal Stem Cells through PI3K/Akt/GSK-3beta/beta-Catenin Signaling Pathway. Biomed. Res. Int..

[55] Fujimori K. (2012). Prostaglandins as PPARgamma Modulators in Adipogenesis. PPAR Res..

[56] Shin S., El-Sabbagh A.S., Lukas B.E., Tanneberger S.J., Jiang Y. (2020). Adipose stem cells in obesity: Challenges and opportunities. Biosci. Rep..

[57] Choe S.S., Huh J.Y., Hwang I.J., Kim J.I., Kim J.B. (2016). Adipose Tissue Remodeling: Its Role in Energy Metabolism and Metabolic Disorders. Front. Endocrinol..

[58] Hajer G.R., van Haeften T.W., Visseren F.L. (2008). Adipose tissue dysfunction in obesity, diabetes, and vascular diseases. Eur. Heart J..

[59] Kolle S.F., Fischer-Nielsen A., Mathiasen A.B., Elberg J.J., Oliveri R.S., Glovinski P.V., Kastrup J., Kirchhoff M., Rasmussen B.S., Talman M.L. (2013). Enrichment of autologous fat grafts with ex-vivo expanded adipose tissue-derived stem cells for graft survival: A randomised placebo-controlled trial. Lancet.

[60] Hong K.Y., Yim S., Kim H.J., Jin U.S., Lim S., Eo S., Chang H., Minn K.W. (2018). The Fate of the Adipose-Derived Stromal Cells during Angiogenesis and Adipogenesis After Cell-Assisted Lipotransfer. Plast. Reconstr. Surg..

[61] Kuhlmann C., Schenck T.L., Tluczynski K., Aszodi A., Metzger P., Giunta R., Wiggenhauser P.S. (2021). Experimental approach to nasal septal cartilage regeneration with adipose tissue-derived stem cells and decellularized porcine septal cartilage. Xenotransplantation.

[62] Taha S., Saller M.M., Haas E., Farkas Z., Aszodi A., Giunta R., Volkmer E. (2020). Adipose-derived stem/progenitor cells from lipoaspirates: A comparison between the Lipivage200-5 liposuction system and the Body-Jet liposuction system. J. Plast. Reconstr. Aesthet. Surg..

[63] Wound Healing Assay Using the ibidi Culture-Insert 2 Well in a µ-Dish 35 mm. https://ibidi.com/img/cms/support/AN/AN21_Wound_Healing_Assay.pdf.

[64] Suarez-Arnedo A., Torres Figueroa F., Clavijo C., Arbelaez P., Cruz J.C., Munoz-Camargo C. (2020). An image J plugin for the high throughput image analysis of in vitro scratch wound healing assays. PLoS ONE.

[65] Wiggenhauser P.S., Kuhlmann C., Blum J., Giunta R.E., Schenck T. (2020). Influence of software parameters on measurements in automatized image-based analysis of fat tissue histology. Acta Histochem..

